# Leptin in Anorexia and Cachexia Syndrome

**DOI:** 10.1155/2012/287457

**Published:** 2012-02-08

**Authors:** Diana R. Engineer, Jose M. Garcia

**Affiliations:** ^1^Division of Diabetes, Endocrinology and Metabolism, Michael E DeBakey Veterans Affairs Medical Center, Houston, TX 77030, USA; ^2^Baylor College of Medicine, 2002 Holcombe Boulevored, Building 109, Room 210, Houston, TX 77030, USA; ^3^Division of Diabetes, Department of Medicine, Endocrinology and Metabolism, St Luke's Episcopal Hospital, Houston, TX 77030, USA; ^4^Huffington Center of Aging, Baylor College of Medicine, Houston, TX 77030, USA

## Abstract

Leptin is a product of the obese (OB) gene secreted by adipocytes in proportion to fat mass. It decreases food intake and increases energy expenditure by affecting the balance between orexigenic and anorexigenic hypothalamic pathways. Low leptin levels are responsible for the compensatory increase in appetite and body weight and decreased energy expenditure (EE) following caloric deprivation. The anorexia-cachexia syndrome is a complication of many chronic conditions including cancer, chronic obstructive pulmonary disease, congestive heart failure, chronic kidney disease, and aging, where the decrease in body weight and food intake is not followed by a compensatory increase in appetite or decreased EE. Crosstalk between leptin and inflammatory signaling known to be activated in these conditions may be responsible for this paradox. This manuscript will review the evidence and potential mechanisms mediating changes in the leptin pathway in the setting of anorexia and cachexia associated with chronic diseases.

## 1. Introdiction

Leptin was discovered in 1994 by Friedman and colleagues after cloning an obese (OB) gene responsible for obesity in *ob/ob* mice [1]. It is a 167 amino acid peptide produced by adipocytes and it is a member of the adipocytokine family. Leptin has been noted to play a major role in body mass regulation by acting in the central nervous system to both stimulate energy expenditure and decrease food intake [2–4]. Named after the Greek word* leptos*, meaning lean, leptin was the first adipocyte-secreted hormone discovered, proving the active role of adipocytes in metabolic signaling.

Leptin crosses the blood-brain barrier in a process that is highly regulated [5–8] and its receptors are found both centrally, in the hypothalamus, and peripherally, in pancreatic islets, liver, kidney, lung, skeletal muscle, and bone marrow [9]. Besides its key role on body weight regulation, leptin affects various metabolic pathways, including growth hormone (GH) signaling [10], insulin sensitivity, and lipogenesis [11]. While leptin levels are directly related to adiposity, there are several other factors resulting in individual variability. Leptin secretion is regulated by insulin, glucocorticoids, and catecholamines [3, 12, 13]. Also, females have significantly higher levels of leptin than men, for any degree of fat mass [14]. Along with adiponectin, leptin assists in peripheral insulin sensitization independent of body weight [15–17]. In leptin-deficient (ob/ob) mice, leptin injections led to dose-dependent reductions in serum glucose levels compared to fed ob/ob controls, before any significant change in body weight occurred [1, 18, 19].

Inactivating mutations of leptin or leptin receptor gene result in the body's false perception of starvation and subsequent hyperphagia, decreased energy expenditure, and severe obesity [20, 21]. In the absence of these mutations and presence of diet-induced obesity, increased adipose tissue results in increased leptin levels. A 10% increase in body weight leads to a 300% increase in serum leptin concentrations [22]. These elevated leptin levels should lead to decreased food intake and increased energy expenditure via physical activity and thermogenesis. Although obese humans have high plasma leptin concentrations, these high levels do not result in the predicted decrease in appetite. This was initially thought to be due to leptin resistance. However, an increasing body of evidence now suggests central leptin insufficiency as a mechanism. The conventional idea of leptin resistance was thought to be due to inhibition of leptin signaling pathways in leptin-responsive neurons, defects downstream of leptin receptor, and blood-brain barrier (BBB) transport limitation for leptin [23–27]. Decreased leptin transfer via the BBB does not, however, compromise intracellular signaling in the hypothalamus. Furthermore, centrally administered leptin effectively decreases the rate of fat accumulation, hyperglycemia, insulin resistance, hyperinsulinemia, and progression to metabolic syndrome in obese rodents [28–30]. The validity of the concept of leptin resistance has, thus, been questioned.

Central leptin insufficiency due to dietary and lifestyle changes for extended periods of time has been shown to result in increased fat accrual, decreased energy expenditure, hyperinsulinemia, hyperglycemia, neuroendocrine disorders, osteoporosis, and impaired memory [6, 31, 32]. Leptin permeability across the BBB is modulated by various endogenous factors, including adiposity, daily mealtimes, intrinsic circadian rhythms governing ingestion behavior, and aging [5–8]. Transport of leptin to the brain is reduced by fasting and increased by pretreatment with glucose [33, 34]. Leptin binding proteins in the blood can affect leptin levels available for transport to the brain. For example, hepatic C-reactive protein (CRP), which is increased in obesity, binds leptin and limits leptin receptor binding and transport across the BBB [35, 36]. Central leptin gene therapy in obese mice resulted in multiple benefits, including normoinsulinemia, euglycemia, elimination of fatty liver, increased energy expenditure, and more than doubling of lifespan [32, 37, 38].

Cytokines, such as IL-6 and TNF-*α*, are increased in obesity and correlate with insulin resistance [39]. After three weeks of a very low-calorie diet, IL-6 levels decrease in adipose tissue as well as in serum. Furthermore, IL-6 knockout mice develop obesity at the young age of 6 months [40]. Unlike leptin, IL-6 has higher CSF concentrations than serum in some obese, but otherwise healthy, men [41]. The presence of increased cytokines, in addition to the hyperleptinemia and central leptin insufficiency, seen in obesity appears to play a key role in the pathophysiology of metabolic syndrome.

Taken together, the data suggest that the leptin system may be more efficient in signaling a decrease in fat mass and lack of nutrients (low leptin state) and triggering a compensatory increase in food intake and a decrease in energy expenditure than as a satiety signal when its serum levels are elevated. Moreover, recent evidence suggests that the neurobiology of leptin signaling in obesity appears to involve central leptin insufficiency, as opposed to the previously postulated notion of leptin resistance. Interestingly, central leptin administration and gene therapy has successfully improved energy homeostasis as well as prevented diet-induced obesity and metabolic syndrome in mice.

The arcuate nucleus (ARC), ventromedial (VMH), dorsomedial (DMH), and lateral (LH) hypothalamic nuclei are important regions regulating food intake and energy expenditure. Disrupting lesions in the ARC, VMH, and DMH of rats resulted in hyperphagia and obesity [42]. Moreover, lesions in the LH resulted in decreased food intake [43]. Binding of leptin to its hypothalamic receptors activates a signaling cascade in the ARC that results in inhibition of orexigenic pathways as indicated by decreased mRNA expression of neuropeptide Y (NPY) and agouti-related peptide (AgRP), and stimulation of anorexigenic pathways as suggested by increases in the mRNA levels of alpha-melanocyte-stimulating hormone (*α*-MSH) and cocaine and amphetamine-regulated transcript (CART) [44, 45]. Activation of POMC/CART-expressing neurons by leptin results in release of *α*-MSH, which subsequently binds to melanocotin receptors (MCRs) and leads to anorexia and increased energy expenditure. At the same time, leptin inhibits NPY/AgRP neurons, which stimulate orexigenic responses and directly inhibits POMC neuron expression as indicated by POMC mRNA expression [46, 47]. Interestingly, there is no feedback from POMC neurons to NPY/AgRP neurons, revealing that the default function of the circuit is to promote food intake [48, 49]. Loss of function mutations of the MC-4R, the most important melanocortin receptor (MCR), is the most common genetic etiology of obesity and accounts for 3–5% of severe human obesity [50, 51], highlighting the relevance of this pathway in humans. Leptin modifies postsynaptic action of orexigenic and anorectic signals via the JAK2 (Janus kinase 2)-STAT3 (signal transducer and activator of transcription 3) and PI3K-PDE3B (phosphatidylinositol-3 kinase-phosophodiesterase 3B-cAMP) pathways [52].

Pinto et al. hypothesized that leptin may cause rewiring of the ARC neural circuit when they found that the NPY/AgRP and POMC neurons in ob/ob and wild-type mice differed. Treatment with leptin normalized synaptic density within six hours, even before leptin levels affected food intake. These findings indicate that leptin may also function via neural plasticity in the hypothalamus [53]. Another anorectic hypothalamic pathway has been recently characterized involving the protein nesfatin-1 and it appears to be leptin-independent. Nesfatin-1 targets magnocellular and parvocellular oxytocin neurons as well as nesfatin-1 neurons to stimulate oxytocin release. Oxytocin then activates POMC neurons in the nucleus of the tractus solitaries (NTS) and induces melanocortin-dependent anorexia in leptin-resistant Zucker-fatty rats. Injecting nesfatin-1 was shown to activate the PVN and result in leptin-independent melanocortin-mediated anorexia [54].

In summary, the discovery of ob/ob mice and leptin has provided evidence that there is hormonal communication between adipose tissue and the hypothalamus, regulating food intake and energy metabolism. Leptin controls feeding via the ARC melanocortin system, by altering gene transcription and neural plasticity. The ARC then integrates all the information it receives and accordingly alters feeding and metabolism through hormonal and neural pathways. The hyperleptinemic state of obesity has been associated with leptin resistance or, more likely, central leptin deficiency.

Convincing evidence suggest that one of the main roles of leptin is to signal a state of nutrient deficiency and fat loss. Low leptin levels in this setting will trigger a centrally mediated, compensatory response leading to increased appetite and food intake, decreased energy expenditure, and, ultimately, weight regain. However, this mechanism does not appear to be preserved in most chronic diseases in spite of the weight loss seen. This manuscript will review the evidence and potential mechanisms mediating changes in this pathway in the setting of anorexia and cachexia associated with chronic diseases.

## 2. Anorexia-Cachexia Syndrome

Anorexia, defined as the loss of desire to eat, despite caloric deprivation, is frequently seen in patients with advanced chronic illness [55]. Cachexia, a term derived from Greek *kakos*, meaning bad, and *hexis*, meaning condition, describes a progressive loss of adipose tissue and lean body mass. Increased proteolysis, decreased protein synthesis, and accelerated lipolysis due to high energy demands result in a dramatic decline in lean body mass and fat mass and increase in mortality in this setting [56, 57]. Caloric restriction per se induces a less severe degree of weight loss and a different metabolic pattern characterized by decreased energy expenditure and preservation of lean mass at the expense of fat loss. This suggests that anorexia alone does not cause the extreme weight loss seen in cachexia. Moreover, nutritional support does not reverse cachexia [58]. Clinically, ACS presents with weight loss, decreased appetite, early satiety, muscle atrophy, and weakness. This process has been observed in various illnesses, including cancer, chronic heart disease, pulmonary disease, chronic kidney disease, and aging.

Anorexia-cachexia syndrome appears to be multifactorial, often associated with the underlying disease process, and related to both peripheral and central neurohormonal signals regulating both appetite and energy expenditure. Inflammatory cytokines, such as tumor necrosis factor- (TNF-) *α*, interleukin- (IL-) 1, IL-6, and interferon- (IFN-) *γ* have been postulated to play a key pathogenic role in the decreased food intake and increased energy expenditure seen in most chronic conditions associated with the anorexia and cachexia syndrome (ACS) [59]. Increased cytokines in the hypothalamus enhance serotoninergic tone through tryptophan, resulting in activation of POMC neurons and subsequent anorexia [60]. IL-1 inhibition in tumor-bearing animals has been shown to improve appetite and promote weight gain [61]. The somatomedin pathway, including GH and insulin-like growth factor-1 (IGF-1), stimulates skeletal muscle protein synthesis and is inhibited by inflammatory cytokines [62, 63]. In spite of the devastating effect that ACS has in patients, its pathophysiology is only partially understood and there are no approved treatments for this condition.

## 3. Crosstalk between Leptin Signaling and Inflammation

Leptin receptors belong to the class I cytokine receptor family and have similar structure to the signal-transducing subunits of the IL-6 receptors [64]. Leptin levels decrease with fasting and increase during the postprandial phase afterwards. These changes are directly correlated with changes in hypothalamic interleukin- (IL-) 1*β* mRNA levels, suggesting that leptin has a proinflammatory role centrally [65]. This link between proinflammatory cytokines and leptin has been illustrated well in animal models. Fasted hamsters treated with the cytokines tumor necrosis factor- (TNF-) *α* and IL-1 showed increased levels of both circulating leptin and leptin mRNA in adipose tissue. These increases in leptin were associated with a decline in food intake [66]. This is also supported by experiments where peripheral leptin administration caused hypothalamic inflammation and central injection of IL-1 receptor (IL-1r) antagonist inhibited the suppression of food intake caused by central or peripheral injection of leptin [67]. Mice lacking the main IL-1 receptor responsible for IL-1 actions showed no reduction in food intake in response to leptin [68]. Increased inflammatory mediators have been shown to increase hypothalamic POMC mRNA expression [69]. Administering melanocortin receptor antagonist centrally results in blockade of inflammatory anorexia [70].

Leptin levels are significantly lower in patients with inflammatory states such as cancer [71] despite correction for body fat. These low levels of leptin, however, are not associated with greater appetite or lower energy expenditure, as might be expected. Disturbances in the feedback mechanism in the hypothalamus and/or release of pro-inflammatory cytokines, such as IL-1, IL-6, and TNF-*α*, are thought to be responsible for cachexia in this setting. These circulating cytokines result in insulin resistance, lipolysis, and loss of skeletal muscle mass [72]. IL-1 influences size, duration, and frequency of meals in rats via hypothalamic signaling [73]. Cytokines also suppress gastric production of the orexigenic peptide ghrelin that decreases production of inflammatory cytokines TNF-*α*, IL-6, and IL-1*β*.

Nuclear factor-*κ*B (NF-*κ*B), a transcription factor for inflammation-related proteins, is activated in the hypothalamus of animal models of infection-associated anorexia. These models are created by administration of bacterial and viral products such as lipopolysaccharide (LPS) and HIV-1 transactivator protein (Tat). In vitro, NF-*κ*B activation stimulated POMC transcription, showing the connection of NF-*κ*B in feeding regulation. Hypothalamic injection of LPS and Tat showed reductions in food intake and body weight, while inhibition of NF-*κ*B and melanocortin cancelled these effects. Moreover, hypothalamic NF- *κ*B is activated by leptin and is involved in leptin-stimulated POMC transcription, showing that it may serve as a downstream signaling pathway of leptin [74]. Paradoxically, inflammation is also thought to play a role in obesity [75]. Obesity in both human [76] and animal models [77] has been associated with increased inflammatory markers, including TNF-*α* and IL-6. In rats and mice with diet-induced obesity, inflammation of both peripheral tissues and the hypothalamus was noted [78–81]. Blocking hypothalamic inflammation signaling via pharmacological approach or gene therapy led to a reduction in food intake and lower body weight in these animals [79, 82].

In summary, the evidence suggests that the central effects of leptin in suppressing appetite and increasing energy expenditure via activation of POMC neurons is at least partially dependent upon inflammation. Moreover, inflammation may influence the same pathways affecting appetite and body weight independently of leptin.

## 4. Leptin in Cancer Cachexia

Cancer anorexia-cachexia syndrome (Cancer-ACS) is found in 80% of patients with advanced cancer. It has been shown to decrease performance status, quality of life, response to therapy, and survival [56, 57]. Cancer-ACS may account for up to 20% of cancer deaths [58]. Although the tumor itself is primarily responsible, treatments such as chemotherapy and radiation, and associated conditions such as depression, pain, gastrointestinal obstruction, and taste alterations can also contribute to weight loss [83]. Cancer-ACS appears to be multifactorial, involving tumor-host interactions that result in catabolism overwhelming anabolism.

Leptin is thought to play a major role in the pathophysiology of cancer-ACS. Animal studies have shown that circulating leptin levels are decreased in the setting of tumor-induced cachexia, as expected given the decrease in fat mass seen in this setting [84]. However, mRNA levels of NPY were decreased and for POMC were increased in the ARC, unlike what is seen in caloric restriction where low leptin levels cause activation of NPY and suppression of POMC pathways. Levels of phosphorylated signal transducer and activator of transcription-3 (P-Stat3), a central molecule activated via the leptin receptor signaling pathway, are upregulated in subsets of *α*-MSH and NPY positive neurons that are not responsive to leptin. This pathway appears to be induced by the cytokine macrophage inhibitory cytokine-1 (MIC-1) via activation of the transforming growth factor- (TGF-) *β* receptor II, suggesting a potential alternative pathway through which MIC-1 could regulate appetite independently of leptin. This is also supported by the fact that MIC-1 infusion can induce anorexia and weight loss in leptin-deficient mice [85].

Leptin levels are significantly decreased in cancer cachexia patients compared to both cancer noncachexia and healthy controls [86, 87]. Proinflammatory cytokines, such as TNF-*α*, IL-1, and IL-6, have been proposed to cause cachexia in spite of low circulating leptin due to increased expression of the hypothalamic leptin receptor [88]. This dysregulation of the normal feedback loop in cancer cachexia may explain why a decrease in leptin does not increase appetite or lower energy expenditure in patients with cancer cachexia. Interestingly, leptin was found to be directly associated with appetite and insulin resistance [87], suggesting that these patients are resistant to the orexigenic effects of hypoleptinemia. Leptin also has been postulated as an early marker of disease progression in advanced ovarian cancer [89]. Moreover, in these patients, there was a clear correlation between disease stage and performance status with markers of inflammation, such as IL-6 whereas low leptin levels were more closely associated with tumor stage and IL-6 levels than BMI.

Given the large role of proinflammatory cytokines in cancer cachexia, the use of anticytokine antibodies and cytokine receptor antagonists has been investigated as potential therapies. Unfortunately, despite promising experimental data, clinical trials have not been conclusive. In the Yoshida AH-130 model, anti-TNF therapy partially reversed metabolic abnormalities in cachexia [90]. Clinical trials, however, showed transient improvement at best. In a double-blinded, placebo-controlled, randomized study, pentoxyphylline, which inhibits TNF*α* transcription, failed to show any benefit on cancer cachexia [91]. In small, unrandomized clinical trials, thalidomide, a TNF-*α* inhibitor, has been shown to improve some cancer-ACS symptoms [92]. Anti-inflammatory cytokines, IL-12 and IL-15, have shown some improvement in cancer-ACS in tumor-bearing animals [93, 94]. Mantovani et al. performed a phase II clinical trial with cyclooxygenase-2 (COX-2) inhibitors in patients with cancer-ACS showing a significant increase in LBM, decrease in TNF-*α*, and improvement in overall performance status [95]. Whether these interventions that blocked inflammation had an effect on leptin or its pathway is not known given that leptin levels or changes in its downstream mediators were not reported in these studies.

In summary, cancer anorexia-cachexia syndrome is a major predictive factor of mortality. In both animal and human models, circulating leptin levels decrease in the setting of cancer-ACS. However, this decrease in leptin is not associated with a compensatory increase in appetite and food intake. Animal studies suggest that hypothalamic inflammation may account for the lack of response of leptin targets to the effects of hypoleptinemia. Although cytokines appear to play a major role in the development of cancer-ACS via central and peripheral effects, preliminary studies targeting this pathway have not shown convincing evidence of a beneficial effect.

## 5. Leptin in Chronic Heart Failure-Induced Cachexia

The incidence of chronic heart failure is steadily rising and carries a poor prognosis. Cardiac cachexia is defined as nonedematous weight loss of >6% of previous normal weight observed over a period of >6 months and is associated with poor prognosis [96]. CHF patients with cardiac cachexia have been noted to have a mortality of 50% at 18 months, versus 17% in noncachectic patients [97]. Various factors can contribute to the weight loss seen in CHF-induced cachexia, including malnutrition from medications, metabolic disturbances (i.e., hyponatremia, renal failure), and hepatic congestion; malabsorption from severe heart failure; or increased nutritional requirements. The basal metabolic rate in these patients is increased by 20% [98]. As in other chronic conditions, inflammation has been implicated as a key aspect of CHF-induced cachexia [99].

Some controversy exists regarding leptin levels in cachectic versus noncachectic CHF patients. While many studies show lower leptin levels in cachectic patients [10, 100, 101], as it would be expected with their decreased fat tissue, other studies report normal levels [102]. These differences may exist due to sex distribution and presence of cachexia in selected subjects. When fat tissue was normalized, leptin levels for both cachectic and noncachectic CHF patients were elevated in comparison to non-CHF controls [10]. Several groups have hypothesized that leptin has a cardioprotective role and that this increase in its levels in the setting of CHF may represent a compensatory response rather than simply a marker of fat atrophy [103–105]. This could also explain the fact that circulating leptin levels directly correlate with NYHA class and overall prognosis in this setting. It is also possible that this increase in leptin is at least partially responsible for the weight loss and anorexia in CHF patients since an increase in leptin would lead to activation of the melanocortin system that in turn would cause an increase in energy expenditure and a decrease in food intake [106]. This hypothesis remains to be tested. It is also postulated that hyperleptinemia in these patients may be a result of insulin resistance [107, 108]. Regardless of the reason, this hyperleptinemia in both cachectic and noncachectic CHF patients suggests that leptin-mediated decrease in appetite and food intake is not particularly important in the development of CHF-induced cachexia.

Leptin may also contribute to CHF-cachexia and obesity-related cardiomyopathy by various cardiovascular mechanisms, including increasing sympathetic activity and producing vasodilation by an endothelium-dependent mechanism peripherally. It also promotes inflammation, calcification, proliferation, and thrombosis in the vasculature [109]. Animal models, however, do not show any increase in blood pressure, despite this increase in sympathetic activity [110, 111]. It is hypothesized that this may be due to leptin's vasodilatory effects via unclear mechanisms [109, 112].

The presence of proinflammatory cytokines in this setting suggests that inflammation plays an important role in the pathogenesis of CHF. Increasing levels of TNF-*α*, IL-1, and IL-6, lead to activation of the renin-angiotensin-aldosterone-system, improving renal and organ perfusion early on. However, TNF-*α* also induces apoptosis and activates protein breakdown in various tissues, including striate muscle. It also contributes to endothelial dysfunction and subsequent decreased blood flow to skeletal muscle, which results in decreased exercise endurance and nutrient supply [113]. Plasma TNF-*α* receptor levels in CHF patients have been associated with poor- long and short-term prognosis [114, 115]. Importantly, TNF-*α* increases the expression of leptin [66, 116]. No current therapy is approved to target cardiac cachexia. Standard treatment of CHF including ACE-inhibitors and beta-blockers has been shown to increase weight in CHF patients [96]. However, it appears that increases in leptin in noncachectic patients with CHF are primarily driven by body weight increases [117].

Although CHF is associated with elevated leptin levels, these are closely related to the amount of fat tissue; hence, levels are lower in cachectic individuals compared to noncachectic, CHF controls. This elevation in leptin may be due in part to insulin resistance but a direct cardioprotective effect of leptin also has been proposed. Taken together, the data suggests that leptin's role in this setting is not entirely related to body weight regulation and that the decreased appetite and increased energy expenditure seen in CHF-induced cachexia are more likely due to other factors. There is a scarcity of therapeutic data on patients with CHF-cachexia, so little is known about leptin responses to treatment at this time.

## 6. Leptin in Pulmonary Cachexia

Chronic obstructive pulmonary disease (COPD), including chronic bronchitis, emphysema, asthmatic bronchitis, and bronchiolitis obliterans, is a leading cause of morbidity and mortality worldwide. Cachexia has been reported in 20–40% of COPD patients and is associated with negative prognosis [118]. Although the increased work of breathing may be partly responsible for increased energy expenditure, this alone does not explain the reported weight loss [119, 120]. It is hypothesized that many different pathways are involved in the pathophysiology of COPD cachexia, including anorexia, nutritional deficiency, hypoxia, increased metabolic rate, inactivity, sympathetic upregulation, inflammation, and anabolic hormone deficiency.

Circulating levels of TNF-*α* and TNF-*α* production by peripheral monocytes are increased in patients with pulmonary cachexia [121, 122]. In both animal and human models, endotoxin or cytokine (TNF-*α* or IL-1) administration produces a dose-dependence increase in serum leptin levels [66, 123, 124]. However, COPD patients were reported to have lower leptin levels compared to healthy controls and these levels correlated well with BMI and percentage body fat [125]. Moreover, there is a loss of circadian variation in leptin levels that may represent alterations in the autonomic nervous system tone. Conversely, serum TNF-*α* levels were significantly higher in COPD patients compared to healthy controls and did not correlate with leptin levels [126], suggesting that in pulmonary cachexia, leptin levels are physiologically regulated and are independent of inflammatory markers, such as TNF-*α*.

In addition to the effects of chronic inflammation, it appears that hypoxia plays a major role in COPD-cachexia. Leptin-deficient animals show CO_2_ retention and respiratory depression; leptin administration to these animals increases minute ventilation and improves lung mechanics suggesting that an increase in leptin levels in patients with lung disease may represent a compensatory response to hypoxia [127–130]. Consistent with this hypothesis, elevated leptin has been described in hypoxic patients compared to BMI-matched controls [131]. It has been shown that expression of the human leptin gene is induced by hypoxia through the hypoxia-inducible factor-1 (HIF-1) pathway. The introduction of noninvasive positive airway pressure ventilation (NIPPV) in patients with severe COPD serves to both decrease energy expenditure and hypoxia. Moreover, body weight significantly improved (increased 12%) in patients with severe COPD and cachexia placed on NIPPV for 1 year [132]. This elevation in leptin was reversed by improving hypoxia, although this was associated with a decrease in fat accumulation in some studies that may have accounted at least partially for the changes in leptin.

In summary, pulmonary cachexia likely involves multiple pathways, including hypoxia and inflammation. Circulating leptin levels are decreased in patients with pulmonary cachexia suggesting that the physiologic regulation of leptin is maintained despite weight loss. Hypoxia also appears to play a role in leptin expression via the HIF-1 pathway that is reversible by correction of hypoxia. Furthermore, correction of hypoxia is associated with weight gain in spite of a decrease in leptin levels. These findings are consistent with the hypothesis that leptin plays a role in regulating the respiratory drive besides its usual role as a metabolic sensor.

## 7. Leptin in CKD Cachexia

Chronic kidney disease (CKD) is a common illness associated with a state of chronic inflammation and, oftentimes, cachexia. CKD-associated cachexia is linked to higher morbidity and mortality [133]. Uremic anorexia appears to be multifactorial. High plasma levels of insulin, leptin, and uremic toxins induce MC4-R stimulation to increase energy expenditure and decrease food intake [134]. Leptin levels are significantly elevated in CKD and ESRD patients and are associated with markers of poor nutritional status, such as low serum albumin and hypercatabolism as well as decline in renal function [135]. Serum leptin concentrations have been shown to inversely associate with survival in some studies [136]. In others, leptin was shown to correlate with fat mass rather than independently affecting food intake or mortality [137]. The hyperleptinemia seen in dialysis patients may be due to poor renal clearance, overproduction, or both. It has been postulated that uremic patients may have an acquired leptin receptor disorder resulting in central insensitivity or resistance, similar to obese individuals. Leptin reduces hypothalamic NPY levels and increases sympathetic activity with hyperinsulinemia, resulting in appetite suppression [138]. Supporting this hypothesis is the observation of increased sympathetic activity, via elevated dopamine, norepinephrine, and serotonin levels, found in uremic patients [139]. Elevated serum acute phase reactants, including C-reactive protein (CRP) and several cytokines, most prominently IL-6 and TNF-*α*, are found in CKD patients and may be associated with reduced appetite in dialysis patients [140]. Increased inflammation in renal failure is multifactorial, and possible factors include decreased renal clearance and increased production of proinflammatory cytokines [141, 142]. The mediators of inflammation act on the central nervous system to alter both appetite and metabolic rate [143, 144].

The administration of AgRP to mice with CKD resulted in increased food intake, normalization of basal metabolic rate, and increases in total body weight and lean body mass, independent of caloric or protein intake. Also, studies in db/db mice show that the lack of leptin receptor is protective against CKD-induced cachexia. Furthermore, manipulation of leptin's downstream mediators in the hypothalamus, MC4 by either gene deletion or by using antagonists of its receptor, confirms the relevance of this pathway in mediating anorexia and weigh loss in the setting of CKD [145].

 A cross-sectional study of 217 hemodialysis patients followed for 31 months showed that those in the lowest tertile of ghrelin levels were the oldest and had the highest BMI, and highest CRP and leptin levels. These patients all had increased mortality risk, despite adjustment for age, gender, and dialysis history. Moreover, those in this group with protein-energy wasting had the highest all-cause and cardiovascular mortality risk (hazards ratios 3.34 and 3.54, resp.) [151]. In the setting of CKD, there is the opportunity to manipulate leptin levels not only by administering recombinant leptin but also by removing leptin from circulation using super-flux polysulfone dialyzers. van Tellingen et al. tried such approach and although leptin levels were significantly reduced, no other parameters such as appetite or body composition were examined [152, 153]. Therefore, the effectiveness of this intervention remains unknown.

Taken together, the evidence shows that CKD and ESRD-induced cachexia are associated with poor prognosis. Elevated levels of inflammatory mediators and leptin are likely results of decreased renal clearance and disease-related inflammation. Activation of the melanocortin system by leptin is key in the pathophysiology of CKD cachexia. Further studies to explore the efficacy of therapeutic options, including polysulfone dialyzers to lower leptin levels, are needed to determine the role of leptin in this setting.

## 8. Leptin in Aging

Weight loss in the geriatric population is a strong predictor of morbidity and mortality [154]. Normal aging involves a decline in appetite, decrease in lean body mass, increase in fat mass, and decrease in energy expenditure [155]. Aging has significant effects on energy homeostasis and dysregulation of adipokines, including leptin. These effects appear to be mediated at least in part by a decrease in the tone of the orexigenic AgRP/NPY pathway, an increase of the anorexigenic CART/POMC pathway, and failure of these pathways in responding to caloric restriction.

Elevated circulating leptin levels with decreased hypothalamic leptin responsiveness have been found in both animal and human models of aging [146]. Using a rodent model of aging, Wolden-Hanson showed that caloric restriction in aged Brown Norway rats failed to induce a compensatory increase in appetite after refeeding, unlike what is seen in young animals [147]. Leptin levels are increased in aged animals paralleling changes in fat mass but fail to decrease in response to fasting, suggesting that hyperleptinemia may contribute to this energy balance dysregulation and play a causative role in the poor tolerance of aged individuals to catabolic conditions. Also, leptin resistance has been proposed as one of the alterations seen in the elderly [156]. Hence, hyperleptinemia may be a compensatory mechanism to overcome the impaired leptin action in the brain. Uptake of leptin in the hypothalamus is significantly lower in old animals [157]. In aged rats, leptin administration does not suppress appetite, hypothalamic NPY expression, circulating leptin levels, or ob mRNA levels in white adipose tissue to the same extent as in young animals [148]. Although expression of the leptin receptor was not investigated in this report, others have shown a decrease in expression of the long form of this receptor during aging [157]. Downstream of leptin, abnormalities in other hypothalamic neuropeptides have been reported as well. Transcript mRNA expression of the orexigenic peptides NPY and AgRP decreases and fails to increase in response to caloric restriction [149, 158]; while CART mRNA expression increases with aging. Although response to exogenous AgRP appears to be maintained, response to NPY administration was significantly blunted in aged animals [159].

Previous studies have shown protective effects of fat mass on morbidity and mortality in the geriatric population [160]. Lipoatrophy and lipodystrophy in aging have been associated with dysregulation of adipokines and, subsequently, metabolic derangements such as insulin resistance, dyslipidemia, metabolic syndrome, hypertension, and hyperglycemia. Centenarians, models of health and longevity, had been reported to exhibit preserved insulin sensitivity and intact adipokine profiles. Studying this population revealed that poor prognosis was associated with dysregulated adipokines, including leptin levels inappropriately low for fat mass [161]. Leptin concentrations in 19 elderly patients with protein-energy malnutrition were significantly lower than their age-matched controls. However, others have reported an increase in leptin levels in aged individuals even after adjusting for fat mass [162]. It has also been shown that the there are increased levels of IL-6 and CRP in aging [150]. Studies in obese patients have suggested an association between hypothalamic inflammation and decreased leptin action via persistence activation of the melanocortin system; no studies, however, have been performed in the elderly [69, 163]. Functional status, anthropometry, and serum markers of nutrition and inflammation, including leptin and CRP, in seventy elderly patients versus controls revealed that those with the lowest functional status and highest frailty indices displayed features of cachexia. Moreover, they had low leptin levels, appropriate for their low body fat, as well as high CRP and IL-6 levels [164]. This suggests that the mechanism for cachexia in the elderly may involve disrupted hypothalamic feedback of leptin from the effects of proinflammatory cytokines like other chronic inflammatory states.

 In summary, weight loss in the geriatric population is associated with higher mortality. In normal aging, fat mass is increased and hyperleptinemia arises. Despite these high levels of circulating leptin, however, there appears to be decreased leptin action and subsequently no decrease in appetite, similar to obese individuals. Moreover, a failed hypothalamic response to caloric restriction appears to be responsible for the poor tolerance of the elderly to catabolic stress. Elderly patients with cachexia tend to have elevated inflammatory markers and low leptin levels, both correlating with worsened prognosis.

## 9. Conclusion

**Figure 1:**
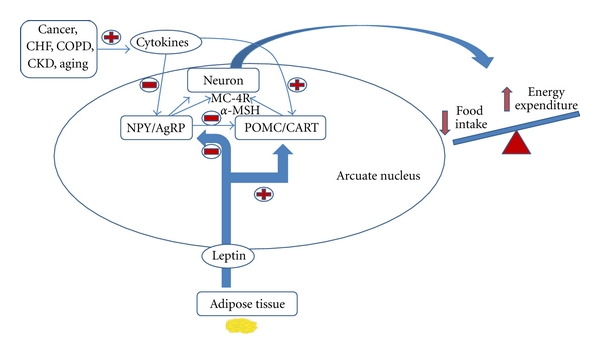
*Summrary of the effects of peripheral hormones on hypothalamic regulation of food intake and energy expenditure.* NPY = neuropeptide Y; AgRP = Agoui-relaed peptide; POMC = pro-opiomelacortin; CART = cocaine-amphetamine-related peptide; *α*-MSH = alpha-melanocyte-stimulating hormone; MC-4R = type-4 melanocortin receptor.

Leptin, a product of the obese gene secreted by adipose tissue, acts centrally to suppress appetite and increase thermogenesis by activating the POMC neurons in the arcuate nucleus and triggering the release of *α*-MSH from POMC axon terminals and subsequently activating MC4-R (Figure 1). Moreover, the NPY and AgRP-producing neurons of the arcuate nucleus, which are suppressed by leptin, can antagonize these anorexigenic melanocortin cells. Consequently, low levels of leptin cause an increase in appetite and reduce energy expenditure.

 Cachexia is a unique process characterized by depletion of adipose tissue and lean body mass found in various chronic diseases often accompanied by anorexia. Anorexia-cachexia can be seen in cancer, CHF, COPD, CKD, and aging (Table 1). All of these conditions are associated with elevated inflammatory markers such as TNF-*α*, IL-6, IL-2, and IL-1*β*. These inflammatory markers may regulate hypothalamic feedback mechanisms and are thought to contribute to the development of cachexia. Leptin receptors belong to the class I cytokine family and there is crosstalk between leptin signaling and inflammation. This crosstalk could explain why, despite low levels of leptin in chronic inflammatory processes such as cancer, COPD, and aging, patients do not have the expected increased appetite or lower energy expenditure.

 Cancer, COPD, and aging-associated cachexia are all associated with low leptin levels, in spite of low appetite and elevated energy expenditure suggesting a state of resistance to the effects of hypoleptinemia. On the contrary, elevated levels have been noted in CKD- and CHF-induced cachexia. In CHF-induced cachexia, the reason for this elevation is unclear but there is no association with weight or fat mass change or with appetite suggesting that leptin may have a different function in this setting and that it likely does not play a major role in the ensuing cachexia. In CKD and ESRD, circulating levels of leptin and inflammatory agents are likely elevated due to poor renal clearance but there is no association with the degree of weight loss or anorexia.

 Given the key role that inflammation appears to play in the pathogenesis of leptin-mediated cachexia, therapeutic intervention with anti-inflammatory drugs may prove to be beneficial in restoring sensitivity to the effect of hypoleptinemia in ACS. COX-2 inhibitors have shown some promise in patients with cancer cachexia. In the setting of uremic cachexia, polysulfone dialysers decrease leptin levels but more studies are needed to evaluate the effect of this intervention on appetite and weight parameters. As we gain more insight into the pathophysiology of cachexia, the therapeutic possibilities increase. Further investigations into anti-inflammatory drugs, appetite stimulants, and immunomodulators in these various conditions are warranted.

## Figures and Tables

**Table 1:** Summary of markers of appetite regulation in various cachectic states.

Condition	Appetite	Body Weight	Circulating Leptin Levels	POMC/*α*-MSH hypothalamic levels	NPY/AgRP hypothalamic levels	Circulating inflammatory markers	Hypothalamic inflammatory markers	References
Cancer cachexia	↓^∗#^	↓^∗#^	↓^∗#^	↑*	↓*	↑^∗#^	↑*	[60, 84–87]
CHF-induced cachexia	↓^∗#^	↓^∗#^	↑^∗#^	unknown	unknown	↑^∗#^	unknown	[10, 99–102, 115]
Pulmonary cachexia	↓^∗#^	↓^∗#^	↓^∗#^	unknown	unknown	↑^∗#^	unknown	[121, 122, 124–126]
CKD cachexia	↓^∗#^	↓^∗#^	↑^∗#^	unknown	unknown	↑^∗#^	unknown	[135, 137, 139, 140, 142, 145]
Aging cachexia	↓^∗#^	↓^∗#^	↓^∗#^	↑*	↓*	↑^∗#^	unknown	[146–150]

^#^—supported by human model data; *—supported by animal model data.
